# Civil society in global health policymaking: a critical review

**DOI:** 10.1186/s12992-018-0393-2

**Published:** 2018-07-25

**Authors:** Eduardo J. Gómez

**Affiliations:** Department of International Development, King’s College London, Room 4.13 North East Building, Bush House, London, W2R 2LS UK

**Keywords:** Civil society, Activists, International health organizations, Policymaking process, Comparative analysis and methodology

## Abstract

**Background:**

A social science approach to the study of civil society’s role and influence in global health policymaking is a new area of scholarly research. In this article, I conduct a critical literature review to assess the recent research done on this topic.

**Main body:**

I find that most research has been narrowly focused on the agenda-setting and policy implementation stages, failing to account for *all* stages of the policymaking process and civil society’s role in it. Additionally, very little effort has been made to test and develop theoretical and analytical policymaking frameworks, clearly and consistently defining and conceptualizing civil society’s role and influence in global health policymaking, provide methodological specificity and diversity, while emphasizing the importance of causal mechanisms.

**Conclusion:**

I conclude by encouraging scholars to address these lacuna in the literature and to explore the utility of political science theory and alternative policymaking models to better define and explain the complexity of civil society’s role and influence in global health policymaking processes.

## Background

In recent years, social scientists have paid increased attention to the role and influence of civil societal actors, such as activists, non-governmental organizations (NGOs), philanthropists, and multilateral agencies in creating and implementing health policy. Most studies have focused on the impact that these actors have on domestic health policy and governance. For the most part, this research has reflected an interest in understanding how transitions to democracy and participatory institutions shapes the provision of equitable and effective healthcare services, as well as the role that civil society plays in designing and implementing policy [22, 25, 46, 62].

In this article, I define civil society as non-governmental actors that create and participate in formal organizations and informal movements with the goal of unifying, expressing, and deepening their normative beliefs and policy interests, while providing a platform from which to engage in public advocacy, collective mobilization, and pressures for issue awareness, policy reform and implementation. I agree with Sullivan [73] in underscoring the diversity of civil societal actors in my definition, such as formal non-governmental organizations (NGOs), faith-based groups, trade unions, charity-based organizations, and pro-business associations. Alternatively, for example, informal movements include social health movements, which do not have formal organizational and financial resources, are united through particular ideological, political, and policy beliefs, and often establish networks of supporters based on these beliefs. As Tarrow [74] explains, these movements often arise to engage in public protest for political and/or policy reform, taking advantage of crisis situations. Therefore, my definition is in large part reflective of the vast literature discussing different aspects of civil society and their involvement in the policymaking process.

Nevertheless, scholars know less about the extent to which civil society has influenced the policymaking processes at the *global level*. While many have critically examined civil society’s role in the domain of global environmental, trade, and human rights policy [31, 41, 50, 64, 77], no concrete effort has been made to examine this issue in the realm of global health policymaking *within* international health organizations. In order to address this lacuna in the literature, I conduct a critical literature review. My overall purpose in this review is twofold: first, to explore what the recent literature has said and understood about civil society’s role and influence throughout the *entire* policymaking process—i.e., from agenda-setting to policy implementation—within international health organizations; and second, to examine the extent to which researchers have applied rigorous social science methodology, such as clear and consistent conceptual definitions, a thorough discussion of research methods, and the application of theoretical frameworks and development.

My findings suggest that the literature is narrowly focused on two aspects of the policymaking process: agenda-setting (primarily) and implementation, failing to address *all* stages of the policymaking process, such as civil society’s influence over the design of policy legislation within international health organizations. The research to date has also been narrowly focused on particular health policy issues, such as tobacco control, without conducting a comparative analysis of different types of healthcare sectors. Finally, rigorous social science research is lacking, such as critically engaging the literature to develop theory, alternative analytical frameworks, clear and consistent conceptual definitions, as well as the specification of causal mechanisms.

### Methodology

My goal in this study was to provide a *critical literature review* [2, 15, 30]. This type of review goes beyond simply summarizing the main findings of the existing literature to carefully assess the quality of the articles selected, to find gaps in the existing literature, and to propose new concepts, categories of measurement, and potential areas of theoretical contribution. Building on Ruckert et al. [61], I aim to explore how the literature has explained and understood civil society’s role in the international policymaking process and if it has used rigorous social science concepts, methods, and theorization processes.

### Article search process

When searching for articles to review, I used two well-known on-line search engines: the *Web of Science* and *PubMed*. These search engines were selected because of their extensive journal databases and because of their reputation for storing and reporting global health studies from a social science and health policy perspective.

The first step in my article search process entailed the usage of key conceptual word search terms in the *Web of Science* and *PubMed*. I was careful to only conduct searches in the core collection for these databases and excluded articles that were published before the year 2000. As Table 1 illustrates, my search terms were comprised of a combination of 4 concepts, first “Civil Society,” which comprised the underlying concepts of “NGO or nongovernmental,” “activism” or “activist,” and “advocacy network,” in combination with three other concepts, “Health,” “Policy,” and “Global/International/Transnational.” All four “Civil society” underlying concepts were used, independently, with each of the other three conceptual terms; no other combination of searches with Concepts 2 to 4 were used. I believed that this approach provided a sufficient amount of articles for potential inclusion in my final analysis.^1^

**Table 1:** Conceptual Search Terms

Concept 1: Civil society (underlying search concepts)	And	Concept 2: Health	And	Concept 3: Policy	And	Concept 4: Global/ international/ transnational
“Civil society”		Health		Policy		Global
“NGO” or “nongovernmental”						OR International
“activism” or “activist”						OR Transnational
“advocacy network”						

I stored the results of my search in separate Microsoft Excel files, under each of the four underlying “Civil society” concepts. My search came up with a total of 353 articles from the *Web of Science* and 269 from the *PubMed* search. However, I was careful not to double count for articles across the 4 Concepts that I reviewed. After omitting those articles that were double counted for the *Web of Science* search, which totaled 14, I had a final total of 339 articles. For the *PubMed* search, I omitted 30 that were double counted across the 4 Concepts, which yielded a total of 239 articles.

Next, I created an *Abstract Screening Matrix* to review the abstracts of the articles found and to determine which would be selected for in-depth reading. When reviewing abstracts I used specific criteria for article inclusion, such as the type of article found, e.g., original research or systematic review; whether the article discussed the role or impact of civil society; if it employed public policymaking frameworks; and if it employed social science methods. I assigned numeric values to each of these criteria, e.g., 0 = no, 1 = yes, 2 = unclear. I excluded articles if they provided only brief commentary and meeting reports; did not analyze the role or impact of civil society; did not employ public policy or social science methods; if they focused explicitly on nation states sans reference to the international community; and if they were not published in the English language. Based on these scores, I then selected articles for an in-depth reading. I used this *Abstract Screening Matrix* for each of the 4 underlying “Civil Society” search concepts noted in Table 1. Of the 339 articles from *Web of Science*, I chose 73 for further evaluation; and out of the 239 *PubMed* articles, I chose 91.

When selecting abstracts and articles for further review, I was careful to omit those that were not empirically focused, such as opinion pieces in popular news magazines, newsletters, and medical journals, such as *The Lancet*. I only chose articles that seemed to be guided by empirical research questions and preferably used a social science approach to empirical analysis. I was also careful to only include those abstracts that discussed civil societal actors in a global policy context, rather than studies focused on their domestic roles and policy influence.

Next, I developed a *Post-Abstract Review Matrix* to analyze those articles selected from the *Abstract Screening Matrix*. I employed several screening criteria to determine which articles would be finally selected and receive an in-depth reading, such as if the article: 1) does not analyze civil societal actors as independent and dependent variables in global (not domestic) policy processes; 2) analyzes the influence of global/transnational actors on national-level civil societal actors; 3) analyzes the influence of global/international civil societal actors on global/international policy-making process; and 4) if articles analyze national level civil societal actors on global/transnational policy-making processes? If articles met either criteria 1 or 2, they were excluded from further review. Out of an initial search of 73 from the *Web of Science* and 91 from the *PubMed* search, 24 from the *Web of Science* and 14 from the *PubMed* search, totaling 38 articles, were selected for in-depth reading.

For those 38 articles that remained, after carefully reading them I then asked a series of questions regarding how global/transnational civil societal actors were defined, how they were theorized with respect to their involvement and influence in global policymaking processes, the key lessons learned, and several other questions related to the factors incentivizing civic mobilization and the research methodologies used. The data obtained from these questions was used to conduct my critical literature review.

## Definitions, methodology, and theory development

With respect to providing clear and consistent conceptual definitions of civil societal actors in the global health policymaking process, less than half, 11, of the 38 articles reviewed achieved this goal. Explicit definitions ranged from “international network of public health experts and lawyers” ([59], p.1); “tobacco control civil society groups” ([44], p.5); “global health networks composed of individuals and organizations producing research and engaging in advocacy on a given health problem” ([20], p.1); and “Global tobacco control epistemic community” ([45], p. 2044). The rest of the articles provided implicit and inconsistent definitions.

With respect to methodology, all of the articles reviewed relied on qualitative methods. No effort was made to systematically measure and quantify the presence and impact of civil society in global health policymaking processes. Moreover, only 3 of the 38 articles reviewed, namely Reubi [59], Mamudu et al. [45], and Gneiting and Schmitz [20], elaborated on their research methodologies. These studies provided a thorough discussion of their case study selection, usage of articles, books, archival materials, and interview strategies (ibid).

Finally, only 2 of the 38 articles reviewed incorporated theoretical frameworks into their analysis, demonstrating how their empirical findings contributed to the literature on global health policymaking processes. While Murphy [51] addressed the literature on complex interdependence between civil society and international agencies and the global public policy literature, Mamudu and Glantz [43] compared their findings to the transnational advocacy network approaches emphasized by Keck and Sikkink [31] with respect to civil societal influence on tobacco control regulation. In general, however, the articles reviewed revealed essentially no effort to engage the theoretical literature and to establish new theories and/or analytical frameworks.

## Civil Society in Global Health Policymaking Processes

The policymaking process within international health organizations, and civil society’s roles and influence within it, varies for each organization, shaped by their unique constitutions [76]. While both U.N. (e.g., WHO, UNAIDS, and World Bank) and non-U.N. (e.g., the Global Fund) have executive boards setting the policy agenda and implementing policy through executive directors and/or regional offices [27, 33, 48, 79], their constitutions vary based on the actual voting process required for policy adoption. The WHO requires a majority vote within the World Health Assembly, while voting within the World Bank Board depends on a weighted process based on the number of shares owned by donor representatives [48, 71]. Moreover, these organizations’ constitutions do not permit civil societal actors to have formal representation within their executive boards and representative assemblies—although the WHA does periodically obtain civil society’s recommendations through its Framework of Engagement with Non-state Actors [78]. In contrast, UNAIDS’ constitution does allow for civil societal representation on its Program Coordinating Board, along with international co-sponsors, such as the WHO, UNESCO, and the UNDP [34]. Nevertheless, within all of these UN organizations, civil societal actors are not allowed to formally vote, or have any kind of influence over, agenda-setting, policy design and implementation processes [78]. In contrast, within non-UN organizations, such as the Global Fund, not only does its constitution allow for civil society’s representation on the governing board (along with governments, the private sector, and people living with disease), which sets the policy agenda, in contrast to UNAIDS, the Global Fund Board does allow civil societal organizations to help establish the policy agenda, an equal say and vote on policy, which requires a two-thirds majority vote among all board members [75]. In further contrast to these UN organizations, the Global Fund works directly with the private sector through Local Accounting Firms to monitor and report how grant funding is being implemented, in turn increasing Principle (grant) Recipient accountability to the Board in Geneva [1, 69].

Returning to my empirical analysis, the results from my critical literature review revealed that studies discussing civil society’s influence in the global health policymaking process emphasized particular areas of research. But first, what do I mean by the term policy “influence”? While in general I agree with other scholars’ description of policy influence as the ability of civil society to shape international organizational policy outcomes in line with civil society’s policy preferences and goals [54], I define “influence” in different ways, depending on which aspect of the policy-making process I am examining.

With respect to agenda-setting, I define “influence” as civil society’s ability to affect the prioritization of global health policy ideas by raising attention to neglected healthcare issues, lobbying and providing consultative services to policymakers, while referring to social norms and justice when working with them. First, as Lee [38] and Murphy [50] explain, civil society has agenda-setting influence when they can increase international and domestic attention to neglected policy issues through the publication of empirical data, research and/or personal testimonies, while strategically referring to this information during agency hearings and debates; this helps to inform and positively influence the interests and preferences of executive boards and/or plenary members. Second, civil society wields influence when they collectively meet and lobby these policymakers through agency committees and/or international conferences in order to explain the need to prioritize neglected policy issues while providing consultative services on how best to engage in policy negotiations [50, 54, 66]. Finally, civil society has agenda setting influence when they appeal to social norms and justice when working with policymakers in order to unify their interests and motivate them to pursue neglected policy issues [50].

With respect to policy formulation and implementation, I define civil societal influence as the latter’s ability to be well positioned within decision-making bodies, helping draft laws, vote on them, while displaying discursive influence during their creation. Indeed, civil society is influential when they are constitutionally permitted to be present, or are invited to participate in, executive board and/or plenary body meetings where discussions are being held about the design of policies, while having the right to co-author and vote on legislation ([39, 57]). I also agree with Pallas and Uhlin [54] that civil societal actors are influential when they have access to, as well as the support of, politically powerful state member representatives within international decision-making bodies [39]. But civil society may also display “discursive” influence. That is, when unrestrained by diplomatic discourse, during international meetings NGOs’ targeted criticisms and accusations towards governments’ policy prescriptions and industry can positively influence the formulation of policy by addressing issues that governments are unwilling to address due to diplomatic concerns, such as morality and ethical issues [39]. Civil Society also wields influence when it works closely with international organizations, via direct partnership and/or contracts, to provide healthcare services, while working with them to monitor, collect data, and report government performance in policy implementation [19, 57, 69]. Finally, civil society can also motivate governments to implement policy by reporting their progress to international organizations and to the media, in turn generating reputation-based incentives to ensure policy success [23].

When carefully reviewing the articles selected for in-depth analysis, I found that the issue that drew most scholarly attention was the agenda-setting process, with a total of 31 out of 38 articles discussing this issue. Moreover, civil society was in general reported to be positively influential during this stage of the policymaking process. Indeed, most of the articles reviewed emphasized the successful collective lobbying efforts used by international NGOs and activists to pressure international organizations into prioritizing particular policies. As Chapman [9] explains, these lobbying pressures emerged through international campaigns, such as the “People’s Health Movement,” through public-private partnerships, as Karnak et al. [35] emphasizes, or, as Smith and Shiffman [70] and Nattras [52] claim, by collectively attending international conferences and vocalizing their interests to representatives from international organizations. Alternatively, the work of Mac Sheoin et al. [42], Murphy [51], Mamudu and Glantz [43], and De Souza and Dutta [14] emphasizes how NGOs and activists come together through international networks of NGOs in order to voice their concern and lobby international organizations, through committees, workshops or conferences, to positively influence the agenda-setting process. Several scholars point out that the policy issues addressed by these civil societal actors through these activities ranged from the creation of international tobacco control treaties and regulations, such as the WHO’s Framework Convention on Tobacco Control (FCTC) [39, 43, 44]; access to essential medicines ([9, 51, 52]); malnutrition [11, 35]; maternal and children’s health [70]; environmental health regulations [42]; and HIV/AIDS prevention programs [14].

In addition to lobbying efforts, the work of Lenchucha et al. [39], Mamudu and Studlar [44], and Nattras [52] explains how international NGOs and activists often participated in direct negotiations with WHO policymakers to help prioritize the creation of international treaties and declarations, such as the 2003 FCTC and the 2001 Doha declaration. Alternatively, Smith and Shiffman [70] and Buse et al. [7] explains how civil societal actors work together to establish international norms, such as rights to health, and strategically use these norms as the basis for collectively pressuring international organizations to prioritize policies, such as woman’s maternal health and universal access to HIV medication. The work of Drope and Lenchucha [16] similarly explains how normative collective movements between activists and NGOs have helped to resolve tensions within the WTO and GATT between international health and trade norms. Alternatively, the work of Storeng and Béhague [72] shows how NGOs, such as the *Safe Motherhood Initiative*, framed woman’s rights to health through a quantitative evidence-based approach rather than a right’s based approach when negotiating with policymakers; this processes was facilitated through the presentation of data on the future economic costs associated with the failure to prioritize investing in maternal health.

The work of Forbes [18], on the other hand, underscored other types of agenda-setting strategies that entailed civil societal actors organizing and attending international conferences to present and emphasize scientific research advocating for particular treatment policies, such as the adoption of *microbicides* drugs for HIV prevention for woman at international AIDS conferences. Similarly, Sasser [65] shows how activists often present research at conferences linking the importance of investing in population and reproductive rights with climate change programs. According to Millard et al. [47], other civil societal organizations, such as the *Venture Strategies for Health & Development*, also organized conferences and presented research in order to pressure the WHO into including particular drugs onto their essential drugs list (EDL), such as *misoprostol*, which is used to prevent maternal death during childbirth. Alternatively, research by Ralston et al. [58] finds that groups such as the World Heart Federation (WHF), in cooperation with the NCD Alliance (a consortium of international NGOs and activists), used the publication of their *Global Heart Journal* to increase awareness of the importance of tackling NCDs; this endeavor contributed, they claim, to the UN’s 2011 High Level Meeting on NCDs.

Alternatively, other articles underscored civil society’s ability to strategically use preexisting institutions within international organizations to increase policymaker attention to particular health issues. Lamy and Phua [36] claim that NGOs and activists used their participation within ASEAN policymaking committees to emphasize attention to particular policy issues. Smith et al. [67] and Mamudu et al. [45] also underscore how activists and NGOs have used governing board committees within the Global Fund, for example, and scientific expert committees within the WHO, to gain access to policymakers and to empress the urgency of creating prevention and treatment policies. Finally, the work of Millard et al. [47], Finkle Dan McIntosh [17], and Murphy [51] claims that NGOs strategically use conference panels and workshops organized by the WHO and the World Bank to display data and provide testimony over the importance of including particular medications onto the WHO’s essential drug list, reproductive health matters, and access to essential medicines.

But why do civil societal actors engage in international agenda-setting processes? Several of the articles reviewed emphasized the importance of normative principles, such as human rights and social justice, as factors motivating agenda-setting behaviors. When it came to proposing environmental regulations on chemical plant industries, or the creation of the FCTC treaty, Mac Sheoin et al. [42] and Mamudu and Glantz [43] claim that civil societal groups often based their efforts on principles of human rights and social justice, i.e., fighting for the rights of those negatively affected by these industries. Smith and Shiffman [70], on the other hand, underscore activists’ efforts to establish norms of women’s rights to health as the basis for collectivizing and engaging in agenda-setting processes. Nattras [52] also found that civil societal demands for access to essential medicines, e.g., through the 2001 DOHA agreement, were similarly motivated by principles of access to medicine as a human right. Alternatively, the work of Newman et al. [53] claims that some activists employed principles of sexual and human rights when striving to prioritize several policy sectors in the 2015 Sustainable Development Goals agenda.

Other work has emphasized the international and domestic political conditions shaping international NGO and activists’ ability to engage in these agenda-setting processes. The work of Gneiting and Schmitz [20] claims that the efficacy of international activist networks in health ultimately rested on the willingness of international organizations to work with them; this willingness is often the product of the network’s ability to form a cohesive and effective coalition, as seen with the tobacco industry (ibid). Alternatively, domestic politics mattered: Buckland et al. [6] emphasizes that strong state-civil societal partnerships provides the political and financial support that NGOs need to mobilize through international ‘issue networks’ (i.e., non-state actors sharing ‘mutual aims,’ principles, and technical expertise) advocating for increased access to essential medicines. For instance, when governments are fully committed to helping NGOs achieve their goals, in partnership with them at the global level, and when NGOs have no fear of domestic political repression, they can successfully work with international activist networks, such as the *Medicines transparency Alliance* (MeTA), to achieve their international agenda-setting objectives (ibid).

Policy implementation was the next major theme that emerged from the articles reviewed—discussed in 12 of the 38 articles selected. Interestingly, the majority of the articles underscored efforts by international networks of NGOs and activists to consistently monitor and hold domestic governments accountable for the implementation of policies created within international organizations. As the work of Nattras [52] and Buse et al. [7] explains, these policies ranged from guaranteeing access to essential medicines and HIV/AIDS treatment for women, as established through the recently proposed Framework Convention for Global Health and the Doha declaration. Alternatively, Chapman [9] emphasized how NGOs established “benchmarks” to measure and monitor government commitments to international normative agreements to healthcare as a human right.

Furthermore, Smith et al. [67], as well as Lamy and Phua [36], claim that international NGOs and activists have often acted as “watchdogs,” that is, incessantly monitoring government commitment to implementing international agreements, such as the FCTC regulations. Landon et al. [37] also argue that activists and NGOs were critical for monitoring government and corporate adherence to policy regulations established within the WHO, such as the efforts of the International Baby Food Action Network’s (IBFAN) monitoring of government adherence to regulatory codes for marketing breastmilk substitutes. Alternatively Hawkes [10] finds that other international NGOs have monitored government commitments to regulate the marketing of high caloric fatty foods to children, a policy consensus put forth by the WHO’s 2005 *Global Strategy for Diet, Nutrition, and Physical Activity*. In other instances, Reubi [59] finds that activists and lawyers have worked together to create judicial monitoring and enforceability mechanisms, taking governments to court for their violation of FCTC agreements, while justifying these actions on the basis of human rights violations.

Interestingly activists also found ways to influence policy implementation processes by employing strategies affecting governments’ international reputation, in turn motivating governments to improve their policy commitments. As the work of Landon et al. [37] underscores, this occurred by publishing reports discussing governments’ unwillingness to adequately implement international policy agreements, as seen with the implementation of FCTC regulations. Reubi [59] has made the same argument in his discussion of the regulation of breast-milk substitutes. Alternatively, in an effort to increase government accountability to WTO member states, Smith et al. [67] claims that international NGOs and activists created international awards for those governments most compliant with FCTC regulations while providing “ashtray” awards to those that were not; this ashtray award, also mentioned in the work of Mamudu and Glantz [43], incentivized states to strengthen their commitment to FCTC regulations.

Nevertheless, despite their innumerable attempts to influence the international agenda-setting and policy implementation process, when it came to the actual formulation of policy, i.e., influencing the design of, and voting on, legislation within international organizations, my review found that civil societal actors were not as influential. For example, research by Gonzalez et al. [24] and Lenchucha et al. [39] in fact suggests that due to civil society’s comparatively weaker position within international health organizations, lack of financial resources and in some instances lack of access to policymakers, NGOs and activists very rarely influenced legislative design; the dearth of supportive articles discussing this process lends credence to this notion. Surprisingly, of all the articles reviewed, only one study, published by Patterson and London [55], provided convincing empirical evidence of international civil societal actors’ ability to directly contribute to the writing (though not voting) of policy legislation, such as UNAIDS’ HIV/AIDS legal guidelines on human rights. While other articles made passing reference to civil society’s impact on the policymaking process, such as the work of Lamy and Phua [36], no causal mechanisms and supportive empirical evidence was provided.

However, the dearth of studies documenting and providing evidence of civil society’s ability to influence policy formulation processes may also reflect the challenges of providing such evidence in the first place. In the literature, very little attention has been paid to documenting precisely *how* civil society affects policy design and outcomes [40]; this is often attributed to scholars’ lack of interest in this issue, deciding instead to provide evidence about causal factors informed by international relations and public policy theory (ibid). Furthermore, the sheer complexity of the policymaking process, which entails several policymaking steps, actors, and interests, has challenged researchers’ abilities to provide evidence (ibid). What’s more, Keck and Sikkink [31] have argued that the multiplicity of activists and NGOs involved can make it difficult to trace and document their influence on policy formulation.

## Discussion

My critical literature review revealed several key lessons and future areas of research. Nevertheless, it is important to first acknowledge the limitations of my review. I realize that my initial search terms were restricted to key concepts, e.g., “Civil Society,” “NGO or non-governmental,” “activism,” “activists,” and “advocacy network.” I restricted my analysis to these concepts in the belief that they would be sufficient for finding articles discussing these and other related civil societal concepts. Nevertheless, my approach placed too much faith in what the articles would reveal, which is problematic considering that even if my articles discussed civil society’s involvement in global health policymaking, it would not capture the myriad of different types of civil societal actors involved in this process. Therefore, my search terms could have been broadened out to include several other groups in “civil society,” such as church associations, trade unions, business groups, charities, and the like. Doing so could have provided more articles covering a broader spectrum of civil societal involvement in global policymaking. Furthermore, I could have also combined these alternative terms with my initial search terms to find more articles discussing the complex interaction of different types of civil societal actors involved in global policymaking.

But these challenges also have implications for my overall findings. First, my restricted search may have overlooked articles addressing factors that I claim the literature has inadequately addressed, such as civil society’s role in policy formulation, as well as theoretical and methodological development. Indeed, by broadening my search and combining different concepts, e.g., “Civil Society” with “business associations” and/or “church groups,” I could have found articles that more thoroughly address how networks of international and domestic civil societal actors are influencing the drafting of policy legislation and the different strategies used to achieve this. Second, this search process could have led to the discovery of NGOs and/or social movements that have been involved throughout *all* stages of the policymaking process, as well as which governments are committed to ensuring that this continues to occur. Third, this approach could have discovered articles that provide alternative methodological approaches to civil society in the policymaking processes, such as cross-national statistical analysis, with a variety of civil society independent variables classified and coded differently, assessing their correlation with policymaking outcomes. And finally, this alternative search process could have discovered articles that provide examples of more complex policymaking models that combine most or all aspects of the policymaking process, e.g., combining multiple streams (agenda-setting) analysis with the street-level bureaucracy (implementation) literature, in turn discussing the complexity of civil society’s roles and influence; this, in turn, could have provided a more nuanced approach to organizing and explaining my critical literature review.

Despite these limitations with how I went about finding scholarly articles, my critical literature review revealed several limitations with the recent literature. First, more work needs to be done clearly defining civil society at the global level, their particular interests, roles, and strategies in global health policymaking. As mentioned earlier, there are currently too many competing definitions and concepts, with more than half of the articles reviewed paying insufficient attention to defining and conceptualizing what civil society is and how it contributes to *all* stages of the policymaking process—i.e., from agenda-setting, to policy formulation, implementation, and evaluation. Those studies providing a clear definition of civil society (11 out of 38 articles reviewed) reflected scholars’ tendency to focus only on particular stages of the policymaking process, such as agenda-setting versus implementation; these differences were often fueled by the interest to apply and test different types of theoretical frameworks, e.g., constructivism and/or realism in international relations theory, to particular stages of the policymaking process. Consequently, and as my critical literature review revealed, there were several different types of definitions and arguments about what civil society is, its role and influence in global health policymaking.

Nevertheless, going forward, scholars should strive to provide a clearer, all-encompassing definition of what civil society is and how it influences *all* stages of the policymaking process. In so doing, researchers will be able to apply and examine the conceptual definition’s applicability and effectiveness in explaining civil society’s roles and influence within several different types of international organizations—e.g., U.N. versus non-U.N.; such a definition, moreover, could also help to better understand which particular stages of the policymaking process civil society is most influential in and why. Finally, providing an all-encompassing definition is important because it confirms recent observations that civil society is becoming influential in all aspects of the global policymaking process [12, 56], and that it is perhaps misleading and limiting to focus on civil society’s influence in only one or two aspects of this process. Such an approach could also educate policymakers within international health organizations and motivate them to take civil society more seriously when devising and implementing policy.

Second, my review underscored the absence of theory development. None of the articles examined strove to propose and examine hypotheses about civil society’s role in global health policymaking, or, alternatively, sought to evaluate preexisting policymaking frameworks and theories with new empirical evidence at the global level.

Going forward, in the area of agenda-setting processes, scholars may consider applying and building on analytical frameworks discussing domestic agenda-setting, such as the multiple streams, policy diffusion, and/or punctuated equilibrium frameworks, approaches that are heavily influenced by political science theory [5, 26, 32, 63, 68]. The causal variables emphasized in these frameworks, such as multiple streams’ emphasis on the importance of policy entrepreneurs coupling the problem, policy, and politics streams, not only helps to highlight the utility of applying political science theory to account for how, when, and why actors influence agenda-setting processes [68], but also which specific actors are involved in this process—i.e., civil society, bureaucrats, or perhaps both. Moreover, this approach helps to explain policy entrepreneurs’ network and coalition building capabilities. Applying these frameworks will also require greater elaboration on the causal mechanisms involved in this process and the empirical evidence substantiating claims.

Similarly, the aforementioned literature discussing civil societal actors’ ability to positively influence policy implementation could benefit from integrating the literature on domestic policy implementation. There is, for example, an extensive literature discussing the importance of domestic NGOs working as policy watchdogs, either on their own or contracted by government officials to hold local governments accountable for effective policy implementation [29, 60]; the mechanisms and strategies employed in this literature could be applied and evaluated at the global level, providing further insight into how civil societal actors monitor and hold governments accountable. Recent work by Smith et al. [69] on NGOs’ monitoring and evaluation strategies within the Global Fund lends credence to the notion that such an approach could work at the global level. Moreover, this implementation literature could be combined with analytical frameworks emphasizing the importance of international reputation-building and soft power strategies in constructivist international relations theory [28]. For instance, one could investigate how international NGOs first monitor governments through watchdog processes, followed by publishing reports and/or giving awards that affect a nation’s international reputation in policy development.

It is important to note, however, that there may be challenges to applying the domestic public policy literature to the global level. First, with respect to agenda-setting, applying the multiple-streams literature, for example, with its emphasis on the importance of the policy entrepreneur(s) (in my case, civil societal organizations) in bringing together the problems, policy, and politics stream through designated institutional venues (e.g., committees within agencies) assumes that these institutions and access to policymakers is always present. And yet, as mentioned earlier, international organizations vary considerably in their willingness to provide these institutions—e.g., the WHA provides such venues, while the World Bank does not; this reflects differences in their governance structures.

Furthermore, governance processes are far less predictable and consistent at the global versus domestic level. Executive boards and plenary legislatures may at times decide to either engage or not engage civil societal actors, or even if they do engage, civil society’s views may not always be taken seriously [40]; this is corroborated by the fact that there is little documented evidence to that affect [69]. But this also reflects three challenges: first, the newness of international organizational constitutions/treaties relative to domestic governments; second, the vagueness of the rules determining civil society’s participation in policymaking processes; and third, the low level of transparency and accountability in ensuring executive board/plenary adherence to civil societal involvement. Domestic agenda-setting models, such as multiple-streams, may therefore not be applicable to some international organizations due to the simple fact, as Béland and Howlett [3] point out, that the underlying assumptions and expectations behind these models were based on older, more stable and transparent democratic institutions, primarily in the Western region of the world.

I may find similar limitations with the domestic policy models focused on implementation processes. For instance, the street-level bureaucracy approach to policy implementation may not apply at the global level, as this framework is based on the assumption that local bureaucrats have a great deal of agency, autonomy, and at times have conflicting interests and beliefs with their central and/or state government managers [4, 49], in turn complicating the policy implementation process. However, at the global level, governing boards often have greater control over the agencies and/or contractual bodies hired to monitor and implement policy. For instance, the Global Fund’s LAFs (Local Auditing Firms) and CCMs (Country Coordinating Mechanisms) at the local level are responsible for implementing and monitoring policy; and yet, both are accountable to the central Governing Board in Geneva [69]. This low level of agency and autonomy, in contrast to the prevailing street level bureaucracy literature, can facilitate policy implementation (ibid).

Nevertheless, these challenges in applying domestic policymaking frameworks to the global level in no way suggests that this cannot be achieved. As international organizations solidify their governance processes, and as civil societal organizations and activists become increasingly important policymaking actors within them, future researchers may benefit from applying and using these analytical frameworks to better explain and understand civil society’s role in global health policymaking.

In addition, none of the articles reviewed made an effort to analyze and explain the *entire* policymaking process, that is, conducting a comparative analysis of civil society’s roles in the agenda-setting, legislative design, policy implementation, and evaluation process at the global level, as well as within and across different health sectors. The articles I examined typically focused on one particular aspect of the policymaking process (primarily agenda-setting), while most failed to systematically compare this process across different health sectors—with the notable exceptions of Gneiting and Schmitz [20], Sasser [65], Smith and Shiffman [70], and Smith et al. [67].

Finally, while I have used a “stages heuristic” approach to policymaking when conducting my critical literature review [63], that is, dividing the policymaking process into the agenda-setting, policy formulation, implementation, and evaluation process, I realize that a limitation to this approach is that in reality, policymaking—particularly at the global level—can be much more complex, failing to follow this linear progression in policymaking stages. This challenge has led others to question the efficacy of the stages heuristic approach [8, 13, 63]. While my intention was to use this approach in order to take the first step in exploring and understanding what has been said about the role and influence of civil society in the global policymaking process, I encourage future scholars to take the next step in building on my approach by creating more complex policymaking models, such as unifying multiple streams and policy diffusion agenda-setting analytical frameworks with, perhaps, a street-level bureaucratic approach to implementation. Furthermore, given my global politics and policymaking perspective, I encourage scholars to combine political science, such as historical institutionalism, path dependency, or institutional change theory [21], with policymaking frameworks to better understand, as de Leeuw et al. [13] explains, the “…wicked, multi-level, and incremental nature of elements in this process,” why and how policies fail or succeed, as well as how and when international and domestic institutions, political actors, policymakers, and civil societal actors interact to facilitate or impede policymaking processes (see also [26, 68]).

## Conclusion

The first endeavor of its kind, this critical literature review of civil society in global health policymaking processes has revealed that the literature’s main focus to date has been on addressing issues of agenda-setting and policy implementation, neglecting to thoroughly discuss and provide evidence about society’s influence in policy formulation, such as drafting laws and voting on them. To date the literature also has not addressed civil society’s role throughout *all* stages of the policymaking process within international health organizations. Furthermore, little effort has been made to engage existing policymaking theories, develop theoretical frameworks, provide clear and consistent definitions of civil society’s roles and influence, provide methodological specificity and diversity, while emphasizing the importance of causal mechanisms. Going forward, social scientists will need to address these issues in order to provide a greater understanding about how and to what extent civil society is positively influencing global health policymaking, contributing to social science method, political and policymaking theory, while providing convincing and helpful empirical evidence for social scientists and policymakers.

## Abbreviations

ASEAN: Association of Southeast Asian Nations
FCTC: Framework Convention on Tobacco Control
GATT: General Agreement on Tariffs and Trade
IBFAN: International Baby Food Action Network
ICSF: International Civil Society Framework
MeTA: Medicines transparency Alliance
NCDs: Non-communicable diseases
NGOs: Non-governmental Organizations
UN: United Nations
WHF: World Heart Federation
WTO: World Trade Organization

## Footnotes

1 Nevertheless, I also acknowledge that while my search is focused on specific search terms, more of an effort could be made to include other terms that is consistent with my aforementioned definition of civil society. For example, by including search terms such as “faith-based groups” “trade unions” and “women’s groups,” several additional articles could have been found, providing a broader, more in-depth understanding of how these various civil societal actors may influence global health policymaking. I encourage future researches to address this limitation with my literature review.
